# Type 1 inflammatory choroidal neovascular membrane in a case of viral uveitis - a case report

**DOI:** 10.1016/j.ijscr.2025.111650

**Published:** 2025-07-10

**Authors:** Rakhi D'cruz, T. Anjana, Mathew Shaji

**Affiliations:** a Chaithanya eye hospital and research centre, Kollam, Kerala, India; b Ahalia eye hospital, Kollam, Kerala, India; c Travancore medicity medical college, Kollam, India

## Abstract

**Introduction:**

Inflammatory type 1 CNVM is a severe but uncommon complication associated with posterior uveitis.

**Case presentation:**

We report a case of type 1 CNVM in a 50-year-old man with a documented history of viral ocular infection. Although inflammatory CNVM is frequently associated with posterior uveitis due to tuberculosis and toxoplasmosis, its occurrence secondary to viral uveitis is rare. The patient's medical history included fever followed by viral conjunctivitis accompanied by superficial punctate keratopathy, for which he was undergoing treatment elsewhere. Despite treatment, he continued to experience pain, redness, photophobia, and metamorphopsia. Upon presentation at our centre,one month following the initial symptoms, a diagnosis of posterior uveitis and CNVM was established, which had not been detected during prior ophthalmological examinations. The subsequent administration of intravitreal anti-VEGF agents along with oral corticosteroids resulted in a dramatic resolution of CNVM.

**Discussion:**

To the best of our knowledge, similar case that started with viral conjunctivitis, anterior uveitis and later progressed to posterior uveitis with a type 1 CNVM has not been reported till date. Inflammatory cells released during uveitis can lead to breakage of Bruch's-RPE complex and lead to CNVM. There is no proper consensus in treating inflammatory CNVM.A combination of intravitreal anti-VEGF agents and intravitreal steroids or oral steroids have been tried by various studies.

**Conclusion:**

Unilateral CNVM developed within one month following an episode of posterior uveitis that initially presented as viral conjunctivitis. Early recognition and timely management with intravitreal anti-VEGF therapy combined with systemic corticosteroids resulted in a favorable visual outcome, with no recurrences observed during the follow-up period.

## Highlights

• Inflammatory CNVM is a rare complication[∼2%] associated with viral uveitis.
• We report a case that presented with viral conjunctivitis, later progressed to posterior uveitis and type-1 CNVM
• Even mild viral conjunctivitis need close follow-up if vision complaints occur, requiring complete examination and prompt diagnostic tests.
• CNVM was successfully treated with anti-VEGF agents combined with oral corticosteroids, with no recurrences.

## 1 Introduction

An inflammatory choroidal neovascular membrane is a vision-threatening complication secondary to Chorio-retinal inflammation, releasing angiogenic factors locally, leading to new blood vessels originating from the choroid, breaking through the Bruch's membrane as a neovascular complex beneath the retinal pigment epithelium (type 1 CNVM) [[1], [2], [3]]. These can distort vision and even cause permanent vision loss by exuding serous fluid, blood, or lipids under or into the retina. The inflammatory cells especially leukocytes and macrophages released during uveitis can break the Bruch's-RPE complex and can cause CNVM [1,2]. The prevalence of CNVM secondary to uveitis can occur with different uveitic entities but most common with posterior uveitis and panuveitis [2].

We present a distinctive case of inflammatory choroidal neovascular membrane (CNVM) secondary to viral fever and viral conjunctivitis in a fifty-year-old male, occurring one month post-infection onset. The incidence of inflammatory CNVM secondary to posterior uveitis is 2 %, predominantly reported in young adults [3]. The most frequently documented cases of CNVM associated with posterior uveitis are observed in conditions such as syphilis, toxoplasmosis, and tuberculosis [2].

## 2 Case report

**Fig. 1:**
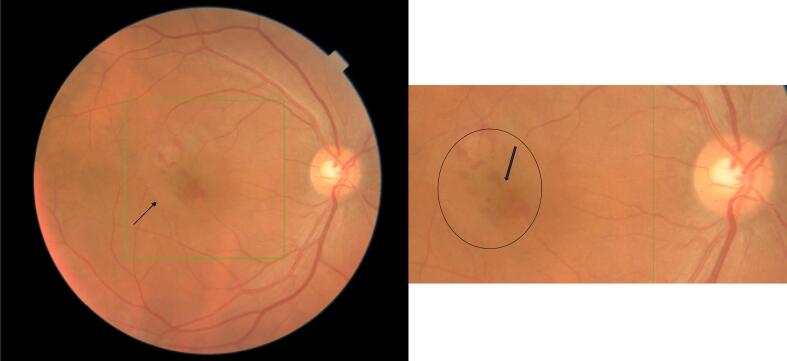
Fundus picture of the right eye showing elevated yellowish lesion superior to fovea with overlying intraretinal hemorrhages and presence of subretinal fluid around 1 disc diameter in circumference surrounding the fovea.

**Fig. 2:**
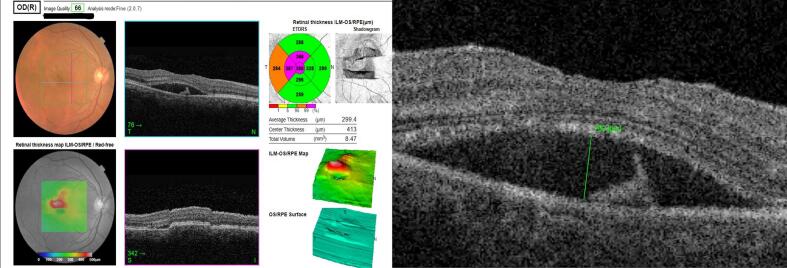
Spectral-domain optical coherence tomography (SD-OCT) image showing features of a Type 1 choroidal neovascular membrane (CNVM). The neovascular complex is located beneath the retinal pigment epithelium (RPE), appearing as an irregular elevation of the RPE with underlying hyperreflective material. Evidence of disease activity is seen in the form of subretinal fluid (SRF) and mild intraretinal fluid (IRF), indicating an exudative response.

**Fig. 3:**
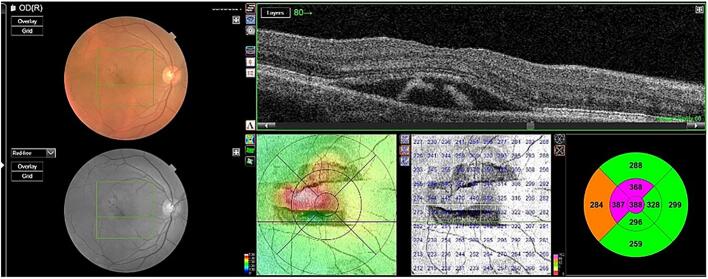
Optical coherence tomography image of the right eye showing presence of subretinal fluid and the presence of hyperreflective lesion corresponding to type 1 CNVM choroidal neovascular membrane, retinal thickening and subretinal fluid.

**Fig. 4:**
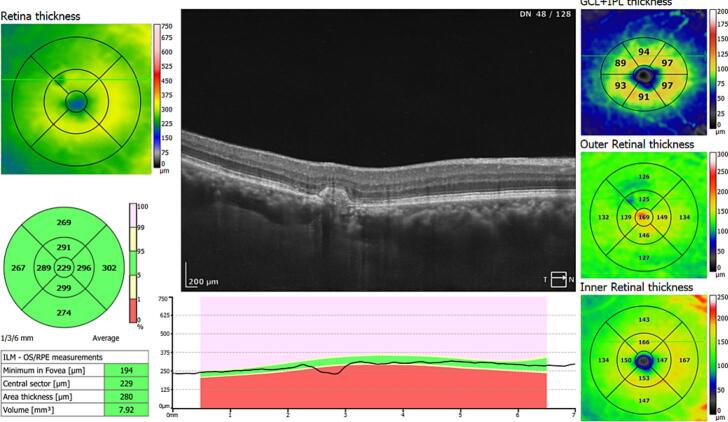
Optical coherence tomography image post injection showing complete resolution of subretinal fluid and scarred choroidal neovascular membrane in right eye.

A 50-year-old male patient presented with a history of fever, unaccompanied by chills or rashes, which was succeeded by bilateral viral conjunctivitis. For the preceding month, he had been receiving treatment at a local ophthalmic hospital, involving lubricants and a combination of antibiotic and steroid medications(moxifloxacin+loteprednol). Despite this treatment, he continued to suffer from redness, lacrimation, and photophobia in the right eye(RE), which proved refractory to the administered medications. The patient reported a diagnosis of elevated intraocular pressure(IOP) in one eye; however, his current prescription did not include any antiglaucoma medications. The patient reported using tablets along with topical medications for approximately two weeks. However, definitive identification of the tablets as acetazolamide or oral steroids could not be established due to the unavailability of prior prescriptions. Furthermore, he reported distorted vision and metamorphopsia in the RE, which had developed over the preceding five days. A similar history of viral conjunctivitis was reported in four family members, though their symptoms resolved more rapidly, within two weeks. The patient's best-corrected visual acuity was 6/18 in the RE and 6/6 in the left eye. Slit-lamp examination of the RE revealed resolving superficial punctate keratitis with subepithelial scars and resolving small-sized keratic precipitates. The anterior chamber exhibited 2+ cells and 1+ flare, a normal iris without pigment disruption or atrophy, a pupil with a brisk response to light, and a clear lens. Posterior segment examination revealed 2+ cells in the vitreous, normal vessels, an absence of vasculitis, and an elevated yellowish lesion superior to the fovea in the macula, accompanied by overlying intraretinal hemorrhages and subretinal fluid extending approximately one disc diameter in circumference around the fovea (Fig. 1). Optical coherence tomography image of the right eye showing presence of subretinal fluid at the Centre of the fovea and the presence of hyperreflective lesion corresponding to type 1 CNVM choroidal neovascular membrane, retinal thickening and subretinal fluid seen superior to fovea (Fig. 2, Fig. 3). Examination of the LE was unremarkable. Intraocular pressure was within normal limits in both eyes. The patient was commenced on prednisolone acetate eye drops, administered six times daily, alongside lubricants. A comprehensive blood evaluation was performed to rule out infectious etiologies, including toxoplasmosis(ToxoIgG,IgM, syphilis(VDRL), sarcoidosis(chest-Xray, serumACE), and tuberculosis(Quantiferon TB gold test). A vitreous tap was scheduled, and an intravitreal anti-VEGF injection was administered in the RE within one week. Viral PCR analysis of the vitreous sample was negative for infectious diseases and viruses. One week postoperatively, resolution of macular edema and improvement in vision were observed. At one month postoperatively, complete resolution of the choroidal neovascular membrane and edema was noted in OCT (Fig. 4), with uncorrected visual acuity improving from 6/9 to 6/6 N6 with correction. The patient was maintained on regular follow-up and completed one year of follow-up without recurrence or decline in best-corrected visual acuity. The case report has been reported in line with SCARE criteria [4].

## 3 Discussion

Inflammatory type 1 choroidal neovascular membrane (CNVM) represents a severe yet infrequent complication associated with posterior uveitis. The etiology of this condition can be both infectious and non-infectious. Among the non-infectious causes are punctate inner choroidopathy (75 %), multifocal choroiditis (33–50 %), serpiginous choroiditis (10–25 %), and Vogt-Koyanagi-Harada syndrome (9–15 %) [3]. The most prevalent infectious causes include toxoplasmosis, West Nile virus, toxocara, histoplasma capsulatum and intraocular tuberculosis [3].

Review by Dhingra et al. states that Leukocyte infiltration and chronic choroidal inflammation are central to CNVM pathogenesis in posterior uveitis. Activated macrophages and microglia release proteolytic enzymes and pro-angiogenic cytokines, disrupting the Bruch's membrane–RPE complex and promoting VEGF-mediated neovascularization, resulting in sub-RPE edema, exudation, hemorrhage, and fibrosis [1].

In our case the atypical progression of fever followed by bilateral conjunctivitis with confluent superficial punctate keratitis (SPK) along with family history indicates a potential viral etiology. The anterior segment findings, characterized by more unilateral involvement with small diffuse keratic precipitates (KP's), minimal anterior segment reaction, and a history of elevated IOP, raise the suspicion of a cytomegaloviral(CMV) etiology, supported by the study by Tereda et al. [5] However, there was evidence of posterior segment involvement, as demonstrated by the presence of vitreous cells, minimal macular edema, intraretinal hemorrhages, and a choroidal neovascular membrane. As the patient presented during the remission phase, one month post-symptom onset, viral markers were negative, which is typical in cases of CMV, as reported by Chiang et al. [6] The specific complaint of metamorphopsia, which developed five days prior to presentation, necessitated a detailed fundus evaluation. Ophthalmologically, the subretinal membrane could have been easily overlooked due to the minimal edema and absence of exudation in this case; the only indirect sign was small intraretinal hemorrhages. Prompt advice to perform OCT facilitated the diagnosis of CNVM. Fluorescein angiography (FFA) and indocyanine green (ICG) angiography were not available at our center and thus were not conducted. To the best of our knowledge, a similar case that began with viral conjunctivitis and anterior uveitis, subsequently progressing to posterior uveitis with a type 1 CNVM, has not been reported to date.

Similar case of a uveitis progressing to CNVM in a case of West-Nile fever has been reported by Zito et al. [7] which required treatment with 3 doses of anti-VEGF, and oral corticosteroids for complete resolution. Another atypical case of herpes virus posterior uveitis with CNVM has been reported by Dicle Hazirolan and Gulten Sungur [8].Another case of CNVM post typhoid fever in a 15-year-old female was reported by Shenoy et al. [9] which was treated with oral steroids alone with good resolution.

A few cases inflammatory CNVM has also been reported in non-infectious causes like juvenile idiopathic arthritis [10] associated uveitis and in immunoglobulinG4 disease [11]. There is no proper consensus regarding the treatment of inflammatory CNVM due to lack of randomized control trials but various management options include observation, intravitreal anti-VEGF injections, photodynamic therapy, systemic corticosteroids and immunosuppressives and laser photocoagulation [3,11,12].

In our case, a single dose of anti-VEGF injection elicited a dramatic response. We also initiated the patient on high-dose oral corticosteroids, which were gradually tapered over an eight-week period. The role of corticosteroids as an anti-angiogenic factor has been demonstrated in inflammatory CNVM [3,11,12]. Most studies recommend a minimum of 3-monthly injections of anti-VEGF, followed by close observation and additional injections as needed [3,12]. However, in our case, there was complete resolution and no recurrence of CNVM during follow-up over the past year. This outcome may be attributed to low inflammation, early diagnosis, and the prompt initiation of high-dose oral steroids, which were slowly tapered to mitigate further inflammatory insult. The primary limitation of our study is the absence of a positive PCR result, which is largely attributable to the patient's late presentation during the remission phase, accompanied by a complication secondary to the inflammatory cascade rather than directly linked to the etiological agent itself. Nevertheless, as is common in cases of viral uveitis, the clinical signs and symptoms are often adequate for making a presumptive diagnosis of the etiological agent, which in our case is suspected to be CMV.

A study conducted by Tereda et al. [5] on the distinguishing features of anterior uveitis caused by HSV, VZV, and CMV indicates that herpetic and varicella-zoster virus infections typically present with more pronounced ocular hyperemia and pain, medium to large keratic precipitates (KP's), sectoral iris atrophy, posterior synechiae, and increased cells and flare in the anterior chamber. The presence of bullous keratopathy, small-sized KP's, coin-shaped KP's, minimal anterior chamber reaction, elevated IOP, diffuse iris atrophy, corneal endothelitis, and endothelial cell loss suggests a CMV etiology.

In the laboratory diagnosis study conducted by Chiang et al. [6] it is indicated that PCR or Goldman-Witmer coefficient (GWC) assays are preferred diagnostic methods. PCR is particularly suitable for detecting viral DNA within the first week of the disease. In contrast, antibodies detected using the GWC may persist throughout the clinical course but are more frequently identified in the later stages of the disease. However, it should be noted that GWC testing is currently unavailable in our region. Another study by Relvas et al. [13] on the efficacy of diagnostic tests for cytomegalovirus uveitis suggests that PCR may not yield positive results if the level of inflammation is low, necessitating repeated sampling if clinical suspicion remains high. In our case, the patient was unwilling to undergo another invasive ocular procedure or further diagnostic tests, which precluded confirmation of the etiological agent.

## 4 Conclusion

Inflammatory type 1 CNVM associated with viral uveitis is very rare. The causes for occurrence can be multifactorial, but a prompt early diagnosis and treatment can lead to favorable visual outcome with less recurrences.

## Consent

Written informed consent was obtained from the patient for publication of this case report and accompanying images. A copy of the written consent is available for review by the Editor-in-Chief of this journal on request. No patient identifying details has been displayed anywhere in the paper.

## Ethical approval

Approval exempt- by institution –Ahalia eye hospital and chaithanya eye institute.

No new methods/ tretaments / any new patient identifying photos /clinical photos used.

The study has a single case report hence excepted from ethical approval, no patient identifying informations are displayed in the paper. Consent has been obtained from patient to use the OCT scan images.

## Funding

None.

## Author contribution

1-Dr Rakhi P D'cruz (RPD); 2- Dr. Anjana T(AT); 3-Dr Mathew Shaji.

a) Conception & design: 1,2 b) Acquisition, analysis, or interpretation of data: 1,2,3 c) Drafting the work or revising it: 1 d)Final approval and accountability for data: 1.

The manuscript has been read and approved by all the authors, the requirements for authorship as stated earlier in this document have been met, and we believe that the manuscript represents honest work.

## Guarantor

Dr. Anjana T.

## Research registration number

N/A.

## Declaration of Generative AI and AI-assisted technologies in the writing process

During the preparation of this work the author(s) used [paperpal, jenni AI] in order to correct language and grammar only in some sections of the case report (case description and discussion). After using this tool/service, the author(s) reviewed and edited the content as needed and take(s) full responsibility for the content of the publication.

## Conflict of interest statement

The author and co-authors have no conflicts of interest.

The study was approved by our ethics committee and adhered to the Declaration of Helsinki.
